# Reanalysis of the effects of “13 Reasons Why”: Response to Bridge et al.

**DOI:** 10.1371/journal.pone.0239574

**Published:** 2020-11-18

**Authors:** Daniel Romer

**Affiliations:** Annenberg Public Policy Center, University of Pennsylvania, Philadelphia, Pennsylvania, United States of America; University of New South Wales, AUSTRALIA

Bridge et al. [1] contest my reanalysis of their data, which they claimed found an increase in suicide in male youth for up to 10 months, beginning the month preceding the release of the first season of the Netflix series, *Thirteen Reasons Why* (13RW). Contrary to their findings [2], my reanalysis failed to detect any increase in suicide in boys beyond the month of the release [3]. I also found an increase in suicide in March, the month prior to the release of the series, and this increase was as large as the one in April that coincided with the release. If one is to argue that a media event such as 13RW had an impact on suicide, one would surely want the effect of the show to follow its release and not to precede it. But Bridge et al. argue that simply promoting a show will have a contagion effect that is comparable in magnitude to the show itself, something that goes far beyond what contagion researchers would propose. And to suggest that the effect resulted from contagion also strains credibility, considering that it appeared only among boys in a show that focused heavily on the death of a young female.

Contrary to the Bridge et al. assertion that the show was heavily promoted prior to its April release, considerable evidence indicates that the show did not create concerns about contagion until April. For example, Niederkrotenthaler et al. [4] also analyzed US monthly suicide data before and after the release of the series. However, those authors used the increase in Twitter discussion about 13RW that occurred in April to focus on that as the month in which contagion might be expected to emerge. Furthermore, Twitter talk declined after May, the second month post series release. And contrary to any hypothesis about the show instigating contagion in March, another study found that there was no noticeable increase in Google searches for suicide-related topics prior to April 6, shortly after the show was released [5]. In addition, Thompson et al. [6] analyzed crisis line discussions before and after the release of the first season. They found no change in trend the month before the release and a sharp *decrease* shortly post the release of the series. Thus, there was no evidence that the series produced anywhere near the attention that would have been required to produce contagion in the month prior to its release and, if anything, the series coincided with a decline in crisis conversations that followed its release.

It is also instructive to examine the results obtained by Niederkrotenthaler et al., which also found effects of the series on males, including a significant increase in young male suicide in March. However, in their analysis of a discrete “pulse” for April (which was similar in approach to my reanalysis), the effect did not exceed the confidence interval for that month, while it did for March. Their analysis of a pulse also did not find an association for females, similar to my analysis. It would seem that both Niederkrontenthaler et al. [4] and Bridge et al. [2] were quick to identify an effect of the series on males when in actuality it did not coincide with the release of the show. All of this goes along with my concern that if one were to make a prediction about contagion effects of the series, it would be for young females. Indeed, Bridge et al. also made this prediction when they noted that “We expected increases in youth suicide rates in both sexes after the series’ release, and that girls would be particularly affected because the show’s protagonist was a teenage girl.” (p. 237). The absence of an effect for females along with an equally strong effect the month prior to the show’s release should have been a sign that something was amiss.

Bridge et al. also argue that their time series analysis “simultaneously accounts for the effects of trend and seasonality.” However, the Holt-Winters model only considers linear trends in its forecasts, and this will seriously underestimate any curvilinear increase in trend, which was especially present for males. The Holt-Winters model essentially produces forecasts based on smoothed values of prior data points. As Diebold ([7], p. 317) notes in regard to this forecasting method, “In practice, of course, we don’t know if the actual data-generating process is close to the one for which the adopted smoothing technique is optimal; instead, we just swallow hard and proceed. That’s the main contrast with the model-based approach, in which we typically spend a lot time trying to find a ‘good’ specification.” Brockwell and Davis ([8], p.318) also warned that the model should be contrasted with other potential models before adopting it as the model of choice for producing forecasts. Indeed, one can see that the Bridge et al. model’s forecast (Fig 1 in Bridge et al., [2]) seriously underestimated the upward trend in overall suicide, and it was especially inadequate in forecasting the more dramatic upward trend in males (S1 Fig in Bridge et al., [2]). Despite what they say, the Holt-Winters model the authors used is hardly state of the art. Recent literature does not even consider this model as a contender in tests of competing approaches to forecasting [9]. It would seem that biostatisticians should consider the use of more recent alternative models when trying to forecast health outcomes.

The approach I took in my reanalysis is much simpler and more defensible than relying on a complex method of smoothing that might not even be better than other approaches that are the state of the art. In my analysis, I approached the problem as a test of an intervention that produces a departure from the underlying components of a series [7, 8]. This requires removing any long-term trends as well as auto-correlation in the series. With those components removed for each gender, one can test for changes in the series that coincide with external events, such as the release of the series. Bridge and Campo claim my analysis was in error because I did not control for seasonality. But I tested for an effect of the show from April to June and found none for either boys or girls, and this was without controlling for seasonality. Examination of the time series after removing trend and auto-correlation did not reveal any seasonal cycles. Thus, failing to adjust for seasonality in my model would, if anything, work in favor of detecting an effect during the spring.

Bridge et al. accuse me of violating the principles of reanalysis proposed by Christakis and Zimmerman [10]. But those principles were directed at conflicts between public health researchers and corporate actors whose reanalyses might be motivated by financial or political interests that are threatened by the findings of the original analysis. As Christakis and Zimmerman stated, …”if the presumption of bias is higher in the reanalysis team, data sharing will more likely impede, not improve, scientific understanding.” (p. 2499). But I don’t see how those concerns apply to my reanalysis. I have no interest other than to provide a more solid scientific account of the possible effects of the Netflix series. If Bridge and Campo have a concern about my scientific bias, they should state that rather than focusing on the question of pre-registration, which they also did not perform, as far as I can tell. In any case, failing to pre-register hardly seems to contradict my findings. I provided all of the analyses that I conducted so that anyone can see how I reached those conclusions.

I also noted in my paper that a more sensitive analysis of US weekly suicide rates might be able to more clearly isolate the effect of the series. Using monthly data may be too insensitive to determine whether the release of the series produced an effect that is different from the rise that was observed in March. One would expect the series to produce contagion with a lag from the date of release and that this would be separable from the increase that was observed in March, prior to the series release.

In sum, as suicide and media researchers, we have the responsibility to report and interpret data that bear on the role of the media without allowing our hypotheses to over-ride the evidence. And given the very crude nature of national suicide rates [11], we are limited in what we can conclude about the extent to which particular media suicide events produced contagion versus protective effects. In view of our still limited knowledge about how these events affect vulnerable audiences, we should resist drawing bold conclusions about effects that defied predictions about both the gender of the victims and the time when the effect should appear.
